# Mr. M'Leod, on the Bulam Fever

**Published:** 1816-03

**Authors:** John M'Leod

**Affiliations:** Surgeon, Royal Navy


*To the Editors of the Medico-Chirurgical Journal & Review.*

GENTLEMEN,

H. M. Ship Alceste, Portsmouth,
January 20, 1816.

THE term *Bulam fever*, which has been lately pressed into our already too complicated catalogue of pyrexial diseases, has, in my mind, (with all due deference to the respectable authorities who use it) a very mischievous effect—conveying the idea that *Bulam fever* is a disease

"*sui generis,*" possessing peculiarities which distinguish it from all others. It has a tendency to confuse and perplex those medical men who have not had experience in tropical complaints. It causes indecision of practice, in a case where decision and promptitude are of all others the most essentially necessary. During the time wasted in a hot climate, settling whether the fever shall be called a Bulam, a Siam, a Jungle, or a Philadelphia one, the patient stands a fair chance of falling a victim to the nosological nicety of his physician. Most unquestionably the general fever *of all hot climates* is susceptible of an infinity of shades and degrees of severity, as it is influenced by locality or idiosyncrasy of constitution; but, from having seen it in almost every quarter where it is capable of existing, I can have no doubt that it is every where *radically the same.* What took place on board the Hankey, I am satisfied would have equally occurred, under similar circumstances (in a crowded undisciplined merchant ship, with a number of unseasoned constitutions on board), had she been at Sierra Leone, Old Callabar, the Gulf of Darien, Vera Cruz, the Havannah, or five hundred other places within the tropics *(or without them)*, where there are heat and noxious effluvia, combining with predisposing causes.

How many hundreds of Guineamen have, from time to time, arrived in the West Indies, from *different* parts of Africa, in the most deplorable state from disease and sickness, but who have not happened, like the Hankey, to be dragged into notice?* What peculiarity can there be in the soil of Bulam (beyond any other marshy or uncleared spot), that *it* should send forth to the world a specific and distinct fever, unlike any ever seen before, and having as distinguishing a character as that of the small-pox? In my humble opinion, it might as reasonably be insisted, that chilblain is the disease of Bergen, because a ship should happen to arrive from that part of Norway with a number of kibe heels on board, although the least reflection will tell us they are the effect of frost and cold

---

* Sir Gilbert Blane, in a paper in the 6th volume of the Medico-Chirurgical Transactions, alleges, as a cause of the *infection* being kept up in the West India islands, this circumstance of the Guineamen coming annually from the African coast. It may be stated as a fact, that not an individual in the West Indies, who is capable of reflection or observation, entertains an idea of the kind. The state of nudity in which the slaves arrive, and the ablutions and oilings which they undergo on removal to the shore, might convince the most decided contagionist, that *they* never carried the "*Bulam*" to the western world.

any where else. The Yellow, the Bulam, the Mediterranean fever, or whatever name *we may fancy* to attach to it, seems to be, in fact, a disease of climate, acting on unseasoned habits, more especially when placed in the vicinity of noxious exhalations; a disease so general, that it cannot *appropriately* derive a name from any particular place; the cure of which, however, may be attempted, in well-disciplined ships or regiments, with fair prospect of success *(but without waiting to classify it)*, by bleeding, the cold affusion, and cathartics; adapting the extent of each remedy, or of all combined, to the circumstances of the case, and by immediately getting to windward of, or removing, if possible, from, the sphere of morbific effluvia—a thing very commonly practised with the most beneficial effects.

Respecting the contagious powers of this fever, I believe it is seldom or ever an *imported* contagion, being of local growth in those places into which it was supposed to be imported, as Cadiz, Gibraltar, and Malaga, for instance: but, like *all fevers*, once *generated*, it, no doubt, will propagate itself, when concentrated within a certain circle, more particularly if that circle is narrowed (as it generally is) by crowding those taken ill into a circumscribed space. I was not at either of the above places *during* the prevalence of the epidemic; but from *subsequent* observation there, and on the authority of a respectable English physician, who was at Malaga in 1804, I can easily conceive, that the extreme fatality might be accounted for on other grounds than that of *imported* contagion. Had the nearest relatives (terrified by unnecessary alarm) not abandoned each other to their fate, on the first appearance of illness; had the physicians and public authorities manfully done their duties, and not deserted their posts; if, instead of treating the sick like mad dogs, hemming them in on one side by quarantine, and on the other by a cordon of fusileers, they had established an airy encampment on the sea side, for the accommodation of the poorer classes, removing them from their dirty unventilated cells, paying a prompt and judicious attention to all at the *commencement* of the complaint; I verily believe that much of the mortality attributed to *imported* contagion might have been prevented.

The non-liability of the human frame to a second attack of this disease, which has been advanced in a late ingenious treatise on the subject, by Mr. Pym, would be a most happy circumstance, could the fact be substantiated. It would lead to very improved arrangements as to the class of men for tropical service. Seamen and soldiers could then be selected, who would be as safe in the West Indies, as a pock-marked subject in a variolous ward. A man having once undergone the fever, is assuredly by no means *so likely* to have a second attack, *whilst he remains in the country*, as a new comer. He is in a certain degree fortified *(by becoming weakened,)* but, let him be transferred to Britain, or other temperate latitude, for a year or two, where he recovers the tone and bracing of that climate; and he returns within the tropics, more especially in early life, as liable to the fever as ever, from carrying back with him the original cause, viz. an unassimilated plethoric constitution.

From non-exposure to the various exciting causes, immediately on his arrival, such as fatigue in the sun, humid neighbourhood, intemperance, &c.; this assimilation may daily and imperceptibly take place, without any convulsion supervening; but until that reduction, or relaxation of habit is completed, the yellow fever hangs over his head.

The cases of Lord Hugh Seymour, and many others, which can be vouched for, on the best authority, are unfortunately proofs too conclusive, of a second occurrence of this fever, *even without leaving the country.*

In giving this account of the disease generally, I state merely, to the best of my judgment, what has fallen under my observation, and not wishing to treat with levity or illiberality the opinions of those who have different ideas of the matter in question. But it is impossible to speak *honestly* on any subject, and not speak *freely*. Every well meaning attempt to promote science, elicit truth, and extend our knowledge of a disease so formidable in a naval and military point of view, is deserving of respect and attention; for, if even found to be incorrect, it does good by promoting discussion, and ultimately leading to improvement.

Should I, in the peculiar service I am now employed on,* or in the course of future experience, have reason to think I have erred in the conception I entertain on this subject, I shall, with the same freedom and fidelity, and with equal publicity, state that error, as I now do my belief, that

* Embassy to China with Lord Amherst.

the *ardent bilious fever* of all hot climates *(Bulam included)* may attack the human frame *repeatedly.*

I am, &c.

JOHN M'LEOD,

Surgeon, Royal Navy.

---

### *Singular CASE of MALFORMATION.*

